# Diagnostic Management of Oncogenic HPV Cervical Infections: The Field Experience in Wuxi, China

**DOI:** 10.3389/fmed.2022.825228

**Published:** 2022-03-22

**Authors:** Yang Liu, Yongxiang Yin, Yi Zhang, Luling Lin, Min Zhao, Qi Chen

**Affiliations:** ^1^Department of Obstetrics and Gynaecology, The University of Auckland, Auckland, New Zealand; ^2^Department of Pathology, Wuxi Maternity and Child Health Hospital Affiliated Nanjing Medical University, Wuxi, China; ^3^Liggins Institutes, The University of Auckland, Auckland, New Zealand; ^4^Department of Gynaecological Cancer, Wuxi Maternity and Child Health Hospital Affiliated Nanjing Medical University, Wuxi, China

**Keywords:** cervical cancer, ThinPrep cytology test, diagnostic accuracy, cervical biopsy, agreement

## Abstract

**Introduction:**

A liquid-based cytology test was introduced for cervical cancer screening in the 2000s worldwide. However, the concordance of diagnostic findings between the liquid-based cytology test and cervical biopsy has not been fully investigated, especially the overall failure rate on the diagnosis of cervical cancer and high-grade squamous intraepithelial lesion (HSIL) by cytology testing. The aim of this retrospective study was to investigate the concordance between ThinPrep cytology and histology test in the diagnosis of cervical cancer and HSIL in HPV-positive women.

**Methods:**

ThinPrep cytology test was performed in 2,472 HPV-positive women. Out of 2,472 HPV-positive women, the cervical biopsy was concurrently performed in 1,533 women. Data on the HPV type and the diagnostic findings of the ThinPrep cytology test and cervical biopsy were collected from our hospital electronic database. The concordance of diagnostic findings between cytology and histology was compared.

**Results:**

The rate of agreement in the diagnosis of the low-grade squamous intraepithelial lesion (LSIL) or HSIL between cervical biopsy and ThinPrep cytology test was 58 or 49%. The overall false negative rate in the diagnosis of cervical cancer and HSIL by ThinPrep cytology test was 6%. However, when considering the total number of HPV-positive women diagnosed with cervical cancer (*n* = 36) and HSIL (*n* = 117) by cervical biopsy, we found that a significant number of HPV-positive women with cervical cancer (*n* = 12, 33%), or women with HSIL (*n* = 77, 66%) were failed to be diagnosed by the ThinPrep cytology test. These HPV-positive women were either diagnosed with cervical infection or ASCUS, or LSIL.

**Discussion:**

Our data demonstrated that in order to ensure an accurate diagnosis, an immediate cervical biopsy in women with cervical infection or ASCUS or LSIL should be strongly recommended in clinical practice.

## Introduction

Cervical cancer is a type of cancer that occurs in the cells of the cervix and is mainly caused by infection with human papillomavirus (HPV). For detection of precancerous lesions and early stages of cervical cancer, screening tests including HPV test and/or conventional Pap test are commonly performed. Due to the limitation of conventional Pap test for detecting cervical dysplasia with a false negative rate of between 5 and 50% (1–4), a liquid-based cytology test such as ThinPrep or SurePath cytology test was introduced for cervical cancer screening in the 2000s worldwide (5). The liquid-based cytology test can improve the diagnostic accuracy of cervical screening resulting in an increased confidence in the reliability of diagnostic findings, compared to the conventional Pap test (6).

Cytologic screening for cervical cancer is based on and corresponds to an underlying carcinogenic process in the development of cervical intraepithelial neoplasia, from squamous atypia, mild dysplasia, moderate dysplasia, severe dysplasia to carcinoma. All these are distinctive precursor lesions of cervical squamous cell carcinoma (7, 8). Negative for intraepithelial lesions or malignancy (NILM), atypical squamous cells of undetermined significance (ASCUS), atypical squamous cells, cannot rule out high grade squamous intra-epithelial Lesion (ASCH), low-grade squamous intraepithelial lesion (LSIL), high-grade squamous intraepithelial lesion (HSIL) and cervical cancer (either squamous cervical carcinoma or adenocarcinomas) are commonly reported by a cytology test (9). However, the comparison of diagnostic findings between the liquid-based cytology test and cervical biopsy, a principal standard for cervical cancer diagnosis is important. Ten to twenty percentage of women with ASCUS by a cytology test showed a varying degree of cervical intraepithelial neoplasia by cervical biopsy (7). A study with a medium sample size evaluated the accuracy of the ThinPrep cytology test in the diagnosis of cervical dysplasia and reported a 74% agreement rate between the ThinPrep cytology test and cervical biopsy. And they also reported an 89.7% positive predictive value of ThinPrep cytology test for diagnosis of LSIL and higher (10). This finding was similar to a recent study with a large sample size on the sensitivity and positive predictive value of ThinPrep cytology test for diagnosis of LSIL and above (11). However, both studies did not evaluate the accuracy of the ThinPrep cytology test in the diagnosis of cervical cancer and HSIL (a precancerous lesion).

Recently many studies have reported that the risk of progression to cervical cancer or HSIL in women with ASCUS or LSIL within 2 years is relatively low (12, 13). In contrast, women with HSIL have a higher potential for progression to cervical cancer and a lower potential for regression (14–17), suggesting HSIL normally requires an immediate biopsy to confirm the cytological finding and to treat aggressively, although not all cases with HSIL would progress to cervical cancer. These studies suggested that colposcopy or an immediate cervical biopsy may not be required in women with ASCUS and LSIL, if the HPV type was not 16 or 18. However, this recommendation should be based on the accuracy of cytology tests in the diagnosis of cervical cancer and HSIL. A recent study with a relatively small sample size (*n* = 131) reported an 86.6% sensitivity of ThinPrep cytology test for diagnosis of HSIL and cervical cancer (18).

About 20% of women who suffer from gynecological cancers including cervical cancer are under 40 years old and they may request fertility preservation [reviewed in (19)]. This consequently requires a more accurate gyanecological cancer diagnosis. Recent studies reported that the combination of ThinPrep cytology test with HPV screening test could increase the sensitivity and accuracy in screening for cervical cancer (20, 21). Therefore, we undertook this retrospective study with a relatively large sample size to evaluate the accuracy of the ThinPrep cytology test in the diagnosis of cervical cancer and HSIL in HPV-positive women, in comparison with cervical biopsy.

## Methods

This retrospective study was approved by the ethics committee of Wuxi Maternity and Child Health Hospital Affiliated Nanjing Medical University, Wuxi, China on (reference number: 202106/1112-22). Due to the nature of the retrospective study, the consent form from patients was not required.

### Study Cohort

From January 2020 to December 2020, 2,472 HPV-positive women who were concurrently examined by a cytology test (ThinPrep cytology test) were included in this study. A study has reported that the accuracy in the detection abnormality of epithelial cells between ThinPrep and SurePath cytology test is similar (22). The management of abnormal ThinPrep cytology test followed the Chinese guideline (Chinese Society for Colposcopy and Cervical pathology) (23). Data on the age of women at the ThinPrep cytology test, the HPV type, and the diagnostic findings of the ThinPrep cytology test and cervical biopsy were collected from our hospital electronic database.

Specimens for the ThinPrep cytology test were taken using cyto-brush by gynecologists and were processed in the Department of Pathology. With the internal control of ThinPrep cytology analysis, all diagnostic results were confirmed by two pathologists. Although an updated guideline indicated that there are 6 categories for reporting cervical cytology test in epithelial squamous cells (NILM, ASCUS, ASCH, LSIL, HSIL and cervical cancer) (9), the diagnostic findings of the ThinPrep cytology test were currently reported as NILM, ASCUS, LSIL, HSIL and cervical cancer (either squamous cell carcinomas or adenocarcinomas) in our system.

The biopsy was performed under colposcopy and the sampling sites were based on the acetic acid reaction assay. Normally, cervical tissues from four sites are used for biopsy. The analysis of the cervical biopsies were performed in the Department of Pathology, Wuxi Maternity and Child Health Hospital Affiliated Nanjing Medical University, Wuxi of China, and all diagnostic results of the biopsy were confirmed by two pathologists.

False negative was defined as women diagnosed with cervical cancer and HSIL by cervical biopsy when they were diagnosed with ASCUS or LSIL by the ThinPrep cytology test. False positive was defined as women diagnosed with no dysplasia by cervical biopsy when they were diagnosed with LSIL or higher by the ThinPrep cytology test. The causes of false negative and false positive results could be interpretation errors due to less experience, and poor technique, including sampling.

### Statistical Analysis

Data was presented as number and percentage. ThinPrep cytology test and cervical biopsy results were arranged in contingency tables, and rates of diagnostic agreement were analyzed by χ2 and McNemar tests using SPSS software. The interrater reliability of agreement between ThinPrep cytology test and cervical biopsy were analyzed using Cohen's kappa. To estimate the accuracy of the ThinPrep cytology test, sensitivity, specificity, positive predictive value, and negative predictive value of the ThinPrep cytology test was calculated.

## Results

A total number of 16,202 women aged over 18 years were examined with an HPV screening test and the ThinPrep cytology test either in our local community hospitals or in our Obstetrics and Gynecology specialized hospital (Wuxi Women' Hospital). Out of 16,202 women, 2,524 women were positive HPV test with at least one of the high-risk types. Out of 2,524 HPV-positive women, 52 HPV-positive women declined further examination: either ThinPrep cytology test or cervical biopsy. The rest of 2,472 women were concurrently examined by ThinPrep cytology test.

**Figure 1:**
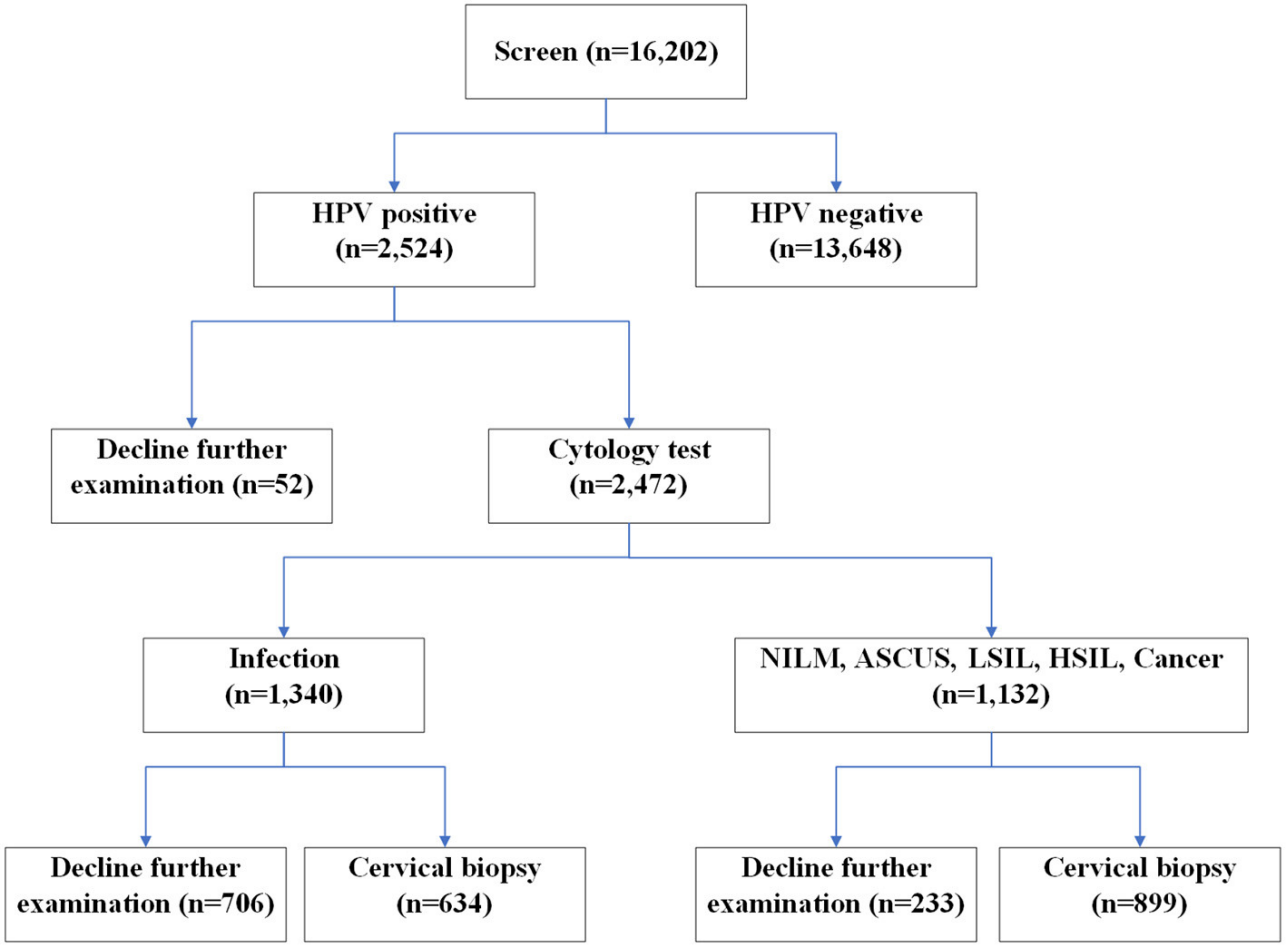
Flow chart of study cohort.

Of the 2,472 HPV-positive women, the ThinPrep cytology test showed that 54% of women (1,340 out of 2,472) were diagnosed with cervical infection. Of the 1,340 women with cervical infection, 47% women (634 out of 1,340) were further examined by cervical biopsy (Figure 1). Furthermore, 46% of HPV positive women (1,132 out of 2,472) were diagnosed with NILM or ASCUS or LSIL or HSIL or cervical cancer. Of the 1,132 women with abnormal ThinPrep cytology results (NILM or ASCUS or LSIL or HSIL or cervical cancer), 79% women (899 out of 1,132) were further examined by cervical biopsy (Figure 1).

The median age of HPV-positive women who were concurrently examined by ThinPrep cytology test was 42 years (ranging from 18 to 82 years). The details of diagnostic findings of the ThinPrep cytology test are summarized in Table 1. 1.6% women (40 out 2,472) were diagnosed with NILM, 24.5% women (606 out of 2,472) were diagnosed with ASCUS, 15.8% women (391 out of 2,472) were diagnosed with LSIL, 3.6% women (89 out of 2,472) were diagnosed with HSIL, and 54% women (1,340 out of 2,472) were diagnosed with cervical infection (cervicitis). Only 6 (0.24%) women were directly diagnosed with cervical cancer (either squamous cell carcinomas or adenocarcinomas), which was further confirmed by cervical biopsy.

**Table 1:** ThinPrep cytology test results in women with HPV positive (*n* = 2,472).

**Cytology test results**	**Number (%)**
ASCUS	606 (24.5%)
LSIL	391 (15.8%)
HSIL	89 (3.6%)
Cancer	6 (0.24%)
NILM	40 (1.6%)
Cervical infection	1,340 (54%)

To evaluate the diagnostic accuracy of the ThinPrep cytology test, we then compared the diagnostic findings of the ThinPrep cytology test with cervical biopsy. Of 2,472 women with the ThinPrep cytology test, 939 women declined cervical biopsy. Seventy five percentage of them (706 out of 939) were diagnosed with cervical infection. 1,533 (61.3%) women were concurrently examined with cervical biopsy (Table 2). The proportions of cases seen in the different rows show that diagnostic results are significantly related (χ^2^ = 678.937 with 9 df, *p* < 0.001).

**Table 2:** The concordance of the results between ThinPrep cytology test and cervical biopsy in women diagnosed with cervical infection, ASCUS, LISL and HSIL (*n* = 1,533).

**ThinPrep cytology test**	**Cervical biopsy outcomes**			
	**Cancer**	**HSIL**	**LSIL**	**NILM**
ASCUS (*n =* 488)	10 (2%)	29 (6%)	186 (38%)	263 (54%)
LSIL (*n =* 334)	1 (0.3%)	14 (4.2%)	**195 (58.3%)**	124 (37%)
HSIL (*n =* 77)	24 (31%)	**38 (49%)**	8 (10%)	7 (9%)
Cervical infection (*n =* 634)	1 (0.16%)	34 (5.4%)	129 (20.3%)	**470 (74%)**

*McNemar test: the diagnoses that define the contingency table are significantly related (χ2 = 52.65, p < 0.001), when ASCUS, LSIL, and cervical infection were grouped as a single category*.

Of the 488 women who were diagnosed with ASCUS by ThinPrep cytology test, 2% (10 out of 488) or 6% (29 out of 488) women were diagnosed with cervical cancer or HSIL by cervical biopsy, respectively. While 38% (186 out of 488) or 54% (263 out of 488) women were diagnosed with LSIL or NILM, respectively (Table 2).

Of the 334 women who were diagnosed with LSIL by the ThinPrep cytology test, 1 (0.3%) woman or 14 (4.2%) women were diagnosed with cervical cancer or HSIL by cervical biopsy. In contrast, 195 (58%) or 124 (37%) women were diagnosed with LSIL or NILM, respectively (Table 2).

Of the 77 women who were diagnosed with HSIL by the ThinPrep cytology test, 24 (31%) or 38 (49%) women were diagnosed with cervical cancer or HSIL by cervical biopsy, respectively. In contrast, 8 (10%) or 7 (9%) women were diagnosed with LSIL or NILM, respectively (Table 2).

Of the 634 women with cervical infection, 1 (0.16%) woman or 34 (5.4%) women were diagnosed with cervical cancer or HSIL by cervical biopsy, respectively. Whereas, 129 (20%) or 470 (74%) women were diagnosed with LSIL or NILM, respectively (Table 2).

The rate of agreement in the diagnosis of cervical infection or LSIL or HSIL between the ThinPrep cytology test and cervical biopsy was 74 or 58 or 49% (Table 2). The Kappa value for showing the interrater reliability was 0.5113. Diagnosis of cervical cancer or HSIL by the ThinPrep cytology test had a sensitivity of 41.3% and specificity of 99%. There were 44% (394 out of 899) of women with ASCUS or higher diagnosed by the ThinPrep cytology test showing cervical infection in cervical biopsy, a false positive rate of 44%. When ASCUS is considered as a cervical abnormality, 11 women with cervical cancer and 43 women with HSIL were not diagnosed by the ThinPrep cytology test. In addition, in women with cervical infection, one woman with cervical cancer and 34 women with HSIL were not diagnosed by the ThinPrep cytology test. Taken together, the false negative rate in the diagnosis of cervical cancer and HSIL by the ThinPrep cytology test was 6%.

When we calculated the total number of women with cervical cancer (*n* = 36) and HSIL (*n* = 117) confirmed by cervical biopsy, we found that 12 (out of 36, 33%) women with cervical cancer or 77 (out of 117, 66%) women with HSIL were incorrectly diagnosed by the ThinPrep cytology test. The overall rate of incorrect diagnosis of HSIL and higher by the ThinPrep cytology test was 58%.

Considering the incidence of cervical cancer and HSIL in the HPV positive in the Chinese population [14% (24)], diagnosis of cervical cancer or HSIL by the ThinPrep cytology test had a negative predictive value of 91.2% and a positive predictive value of 85.9%.

There were 418 cases with HPV type 16 positive, or 163 cases with HPV type 18 positive in total (*n* = 2,524). Of the 418 cases with HPV type 16 positive, 23.6% (99 out of 418) cases were not concurrently examined by cervical biopsy. Of the 163 cases with HPV type 18 positive, 27% (44 out 163) cases were not concurrently examined by cervical biopsy. We then analyzed whether the HPV genotypes could affect the disagreement between the ThinPrep cytology test and cervical biopsy. We found that in those women with HPV type 16 or 18 positive who were concurrently examined by cervical biopsy, there was no association of HPV type 16 or 18 positive with the disagreement of diagnosis between the ThinPrep cytology test and cervical biopsy in the diagnosis of cervical cancer, HSIL and LSIL (Table 3).

**Table 3:** The distribution of HPV 16 or 18 positive in women with disagreement diagnosis between the ThinPrep cytology test and cervical biopsy.

	**Number of disagreement diagnosis**	**HPV type 16 or 18 positive (*n*, %)**	**HPV type 16 or 18 negative (*n*, %)**
ASCUS	Cancer (*n =* 10)	4 (40%)	6 (60%)
	HSIL (*n =* 29)	15 (51%)	14 (49%)
	LSIL (*n =* 186)	55 (30%)	131 (70%)
	NILM (*n =* 263)	44 (17%)	219 (83%)
LSIL	Cancer (*n =* 1)	0 (0%)	1 (100%)
	HSIL (*n =* 14)	9 (64%)	5 (36%)
	LSIL (*n =* 195)	40 (20%)	155 (80%)
	NILM (*n =* 124)	19 (16%)	105 (84%)
Cervical infection	Cancer (*n =* 1)	0 (0%)	1 (100%)
	HSIL (*n =* 34)	13 (38%)	21 (62%)
	LSIL (*n =* 129)	36 (28%)	93 (72%)
	NILM (*n =* 470)	113 (24%)	357 (76%)

In addition, due to the difference in the natural history of cervical intraepithelial neoplasia (CIN) 2 and CIN 3, we also analyzed the number of CIN 2 and CIN 3 in histological HSIL cases who were previously diagnosed with ASCUS and LSIL by cytology (*n* = 43). We found that overall, 16 (37%) histological HSIL cases who were previously diagnosed with ASCUS/LSIL by cytology were CIN 2. In contrast, 27 (63%) histological HSIL cases who were previously diagnosed with ASCUS/LSIL by cytology were CIN 3.

## Discussion

In this retrospective study with relatively large sample size, we found that the rate of agreement in the diagnosis of cervical infection, LSIL, or HSIL between the ThinPrep cytology test and cervical biopsy was 74 or 58 or 49%, respectively. The sensitivity or positive predictive value or negative predictive value of the ThinPrep cytology test for diagnosis of cervical cancer and HSIL was 41.3 or 85.5 or 93.8%, respectively. The false negative rate in the diagnosis of cervical cancer and HSIL by the ThinPrep cytology test was 6%. However, when considering the total number of HPV-positive women diagnosed with cervical cancer and HSIL by cervical biopsy, we found that a significant number of women with cervical cancer (12 out of 36, 33%), or women with HSIL (77 out of 117, 66%) were not diagnosed appropriately by the ThinPrep cytology test.

Liquid-based cytology test such as the ThinPrep or SurePath cytology test has significantly increased the detection rate of ASCUS or LSIL or HSIL and cervical cancer (25). Although a recent study indicated that the SurePath cytology test was associated with the increased detection rate in HSIL, compared to the ThinPrep cytology test (25), other studies reported that there was no difference in the detection rate of ASCUS or LSIL or HSIL and cervical cancer between the SurePath and ThinPrep cytology test (22). The rate of agreement with cervical biopsy has been used to measure the diagnostic accuracy of cytology test. In our current study, we found that the rate of agreement with cervical biopsy in the diagnosis of LSIL or HSIL by ThinPrep cytology test was 58 or 49%, which was relatively low, in comparison with another study showing 74% of agreement on the diagnosis of LSIL and higher (10). The Kappa value for showing interrater reliability was 0.5113, which suggested a weak agreement (26). The positive predictive value of the diagnosis of cervical cancer and HSIL by the ThinPrep cytology test reported in our current study was 85.5%, which is similar to other studies (10, 18). Taken together, our data further confirmed a high positive predictive value of the ThinPrep cytology test in the diagnosis of cervical cancer and HSIL.

In addition to the positive predictive value, false positive rate and false negative rate are also well-used to measure the diagnostic accuracy. In our current study, we reported a 6% false negative rate in the diagnosis of cervical cancer and HSIL, which was slightly lower than another study which showed a 9.9% of false negative rate in the diagnosis of HSIL and higher (10). However, in our current study, we showed a 44% of false positive rate in the diagnosis of LSIL and higher. Our false positive rate was similar to a recent study with a 49.5% of false positive rate in the diagnosis of LSIL and higher, but significantly higher than another study with a 10.3% of false positive rate in the diagnosis of LSIL and higher (10). This could be because our pathologists in the local community hospitals may not be well-trained and may have less experience. They may over-diagnose the cases with cervical infection to avoid misdiagnosis. The higher false positive rate also reflected a lower sensitivity of diagnosis of cervical cancer and HSIL by the ThinPrep cytology test reported in our study (41.3%), compared to other studies showing more than 86% of sensitivity (10, 18).

There is considerable evidence that the risk of progression to cervical cancer or HSIL in women with ASCUS or LSIL is relatively low. To avoid overdiagnosis, some women with ASCUS or LSIL or cervical inflection may not be recommended to perform an immediate cervical biopsy or colposcopy, if their HPV type was not 16 or 18 (Medical Services Advisory Committee. MSAC Outcomes. Application No. 1276 – Renewal of the National Cervical Screening Program. Australian Government Department of Health; 2014). This is also because, to date, there was no study reporting the actual number of women with cervical cancer or HSIL who were not incorrectly diagnosed by the ThinPrep cytology test, if an immediate cervical biopsy or colposcopy was not performed. Although a recent study reported increased sensitivity of diagnosis of the cervical lesion by the combination of ThinPrep cytology and HPV screen test (21), the biopsy is still considered as a gold standard. Our current study also showed a high positive predictive value on the diagnosis of cervical cancer and HSIL by the ThinPrep cytology test. However, in 36 women with cervical cancer, and 117 women with HSIL confirmed by cervical biopsy, 12 (out of 36, 33%) women with cervical cancer, and 77 (out of 117, 66%) women with HSIL were not diagnosed appropriately by the ThinPrep cytology test. The overall failure rate on diagnosis in cervical cancer and HSIL by ThinPrep cytology test was 58%, which was higher than another study showing a 30% of overall fail rate on diagnosis in HSIL and higher (10). Interestingly, this finding was regardless of the HPV genotypes that women were infected with. Due to the small sample size in disagreement, especially in cancer, future study is required to confirm whether the HPV genotypes do not affect the disagreement between the two tests.

Our current guideline recommends a concurrent cervical biopsy when women have abnormal ThinPrep cytology test results and their HPV genotype is 16 or 18, but our current study still showed that 24% or 27% of women with HPV 16 or 18 positive were not concurrently examined by cervical biopsy. The overall incidence of HSIL or cervical cancer in women with HPV positive is ~12% (24) or 2–3%. Therefore, 30% of the failed rate, reported in the other study on the diagnosis of HSIL and higher by ThinPrep cytology test is still relatively high (10). Our data (the Kappa value) also showed a weak agreement between the ThinPrep cytology test and cervical biopsy. Taken together, our data suggest that there is a risk of failing to diagnose cervical cancer and HSIL by theThinPrep cytology test. In addition to the cytology test, in clinical practice, colposcopy is also well-used as a preoperative tool for the detection of inner lesions during routine examination. Colposcopy has been shown to have high diagnostic effectiveness and can be used as a conservation surgery (27, 28).

Currently, our hospital clinical guideline strongly recommends an immediate cervical biopsy or colposcopy in women diagnosed with ASCUS or LSIL by the ThinPrep cytology test, regardless of the HPV genotypes that women are infected with. The frequency of concurrent cervical biopsy in women diagnosed with ASCUS or LSIL by the ThinPrep cytology test was more than 80% in our hospital. Whereas, the frequency of concurrent cervical biopsy in women diagnosed with cervical infection was relatively low (47%). However, in our current study, we found that 34 women with HSIL (30% in total of HSIL cases) were diagnosed with cervical infection by ThinPrep cytology test, which was relatively high. Therefore, an immediate cervical biopsy in these women should be strongly recommended as well.

In our current study, we found a high incidence of ASCUS in our population, compared it to other countries. Although we do not know the exact reason, a recent study found that women with a shorter duration of HPV vaccination had a high incidence of ASCUS, compared to women with a longer duration of HPV vaccination (29). HPV vaccination was only widely introduced in 2016 in China. The HPV vaccination rate is still relatively low. Unvaccinated or shorter time of HPV vaccination may result in a high incidence of ASCUS seen in our current study. Promotion of HPV vaccination should be encouraged as well, in order to reduce the incidence of ASCUS in China.

There are some limitations of our current study. Firstly, this is a retrospective study and there is no data on follow-up. Secondly, there was no data about HPV vaccination. However, due to the majority (95%) women with HPV positive included in this study being aged over 25 years, we then speculate that most of them are not HPV vaccinated. The management of ASCUS/LSIL may also be different between Chinese and other guidelines, suggesting biopsies are not systematically required in some countries if colposcopy is normal and satisfactory. Our updated guideline (2018) suggested a follow-up with colposcopy in women with ASCUS or LSIL diagnosed by a cytology test (22). Finally, we do not have data on ThinPrep cytology test in HPV negative women, which may have a risk of missing women with cervical lesions.

In conclusion, in this retrospective study, our data shows a relatively lower incidence of cervical cancer or HSIL in women with ASCUS or LSIL or cervical infection, confirmed by cervical biopsy. However, without an immediate cervical biopsy, a significant number of women with HSIL (77 out of 117, 66%) or cervical cancer (12 out of 36, 33%) confirmed by cervical biopsy were not appropriately diagnosed by the ThinPrep cytology test. In addition, the most histological HSIL women who were previously diagnosed with ASCUS/LSIL by cytology were CIN 3 in our study cohort. Therefore, although the positive predictive value of the diagnosis of cervical cancer and HSIL by the ThinPrep cytology test is high, to avoid a failure on diagnosis an immediate biopsy in women with ASCUS or LSIL or cervical infection should be strongly recommended in clinical practice, regardless of the HPV genotypes.

## Data Availability Statement

The raw data supporting the conclusions of this article will be made available by the authors, without undue reservation.

## Ethics Statement

The studies involving human participants were reviewed and approved by the Ethics Committee of Wuxi Maternity and Child Health Hospital Affiliated Nanjing Medical University, Wuxi, China on (reference number: 202106/1112-22). Written informed consent for participation was not required for this study in accordance with the national legislation and the institutional requirements.

## Author Contributions

YL: data collection. YZ and LL: data analysis. YY, MZ, and QC: study conceptualization and manuscript drafting. QC: manuscript finalization. All authors were involved in the drafting, editing, and approval of the manuscript for publication.

## Funding

This study was supported by high-level talent training project of Wuxi Taihu Talent Plan (reference number BJ2020072 to YY).

## Conflict of Interest

The authors declare that the research was conducted in the absence of any commercial or financial relationships that could be construed as a potential conflict of interest.

## Publisher's Note

All claims expressed in this article are solely those of the authors and do not necessarily represent those of their affiliated organizations, or those of the publisher, the editors and the reviewers. Any product that may be evaluated in this article, or claim that may be made by its manufacturer, is not guaranteed or endorsed by the publisher.
